# Effects of Consuming White Button and Oyster Mushrooms within a Healthy Mediterranean-Style Dietary Pattern on Changes in Subjective Indexes of Brain Health or Cognitive Function in Healthy Middle-Aged and Older Adults

**DOI:** 10.3390/foods13152319

**Published:** 2024-07-23

**Authors:** Cassi N. Uffelman, Roslyn Harold, Emily S. Hodson, Nok In Chan, Daniel Foti, Wayne W. Campbell

**Affiliations:** 1 Department of Nutrition Science, Purdue University, West Lafayette, IN 47907, USAhodson7@purdue.edu (E.S.H.); chan316@purdue.edu (N.I.C.); 2 Department of Psychological Sciences, Purdue University, West Lafayette, IN 47907, USA

**Keywords:** fungi, *Agaricus bisporus*, *Pleurotus ostreatus*, health-related quality of life

## Abstract

Limited research suggests mushroom consumption may improve indexes of brain health. Mushrooms contain bioactive compounds and antioxidants capable of crossing the blood–brain barrier and impacting vital neurological processes. We conducted a randomized controlled feeding trial assessing the effects of adopting a healthy U.S. Mediterranean-style dietary pattern (MED) with or without mushrooms on indexes of brain health and well-being. Sixty adults (aged 46 ± 12 y; BMI 28.3 ± 2.84 kg/m^2^; mean ± SD) without severe depression consumed a fully controlled MED diet with 84 g/d of mushrooms (4 d/week white button and 3 d/week oyster) or without (control with breadcrumbs) for 8 weeks. At baseline and post-intervention, surveys were used to evaluate anxiety, depression, mood, and well-being, and behavioral tests were used to evaluate cognition. Consumption of the MED diet, with or without mushrooms, increased (improved) self-reported vigor/activity (Time *p* = 0.026) and both behavioral measures of immediate memory (Time *p* < 0.05). Mixed effects were observed for other domains of neuropsychological function, and there were no changes in other measured indexes of brain health with the consumption of either MED diet. Adopting a healthy MED-style dietary pattern, with or without consuming white button and oyster mushrooms, may improve vigor/activity and immediate memory among middle-aged and older adults.

## 1. Introduction

Brain health diseases including psychiatric disorders and dementia affect millions of Americans. Mounting evidence suggests diet is an important mediator in the promotion or prevention of various brain health diseases [1,2,3]. While consumption of ultra-processed foods is associated with mild depression and anxiety [4], consumption of foods with antioxidant and anti-inflammatory properties is associated with a reduced risk of depression and neurodegenerative diseases [5,6,7].

Mushrooms, edible fungi, have a long history of consumption worldwide given their purported health-promoting properties and unique nutrient profile. Primarily demonstrated in observational research, limited evidence suggests a protective role of routine mushroom consumption on symptoms of depression. Individuals in a Korean population who self-reported ≥ one 30 g serving per month of mushrooms (all varieties) had lower depressive symptoms compared with individuals who rarely or never consumed mushrooms [8]. Partly consistent with this, investigators conducting a population-based study using data from the United States National Health and Nutrition Examination Survey (NHANES) reported that participants in the middle tertile (median intake 4.9 g/d) had lower odds of depression compared with non-consumers [9]. While there was no difference between the highest tertile (median intake 19.6 g/d) and non-consumers, when mushroom consumption was divided into the two groups of consumers and non-consumers, mushroom consumption, regardless of the amount, was associated with lower odds of depression [9]. Multiple other studies conducted among cohorts in Asian countries indicate that dietary patterns characterized by high mushroom consumption are associated with lower depressive symptoms [10,11,12,13,14]. This apparent relationship between mushroom consumption and depression is unclear given the results are confounded by other dietary factors, including consuming other healthful foods such as fruits and vegetables.

Distinct from plant- and animal-based foods, mushrooms contain several essential nutrients (e.g., selenium, B vitamins [B2, B3, B5], copper) and bioactive compounds, including the antioxidants glutathione and L-ergothioneine [15,16,17,18]. Notably, mushrooms are the primary dietary source of L-ergothioneine for humans, who are incapable of synthesizing this amino acid [19]. L-ergothioneine has been proposed as an adaptive antioxidant, capable of crossing the blood–brain barrier, in which tissue levels increase in response to oxidative stress through the specific transporter, OCTN1 [20,21,22]. Increases in tissue levels of L-ergothioneine are protective against further damage, including lipid peroxidation and neuronal damage, caused by β-amyloid plaque deposition, a biomarker of mild cognitive impairment [23]. Observational research indicates that whole blood and plasma L-ergothioneine concentrations decline with age, are lower in adults with mild cognitive impairment, and are predictive of cognitive and functional decline, including memory, executive function, attention, visuomotor speed, and language [24,25]. While few randomized controlled trials have been conducted in humans assessing the influence of mushrooms on cognition, the investigators of one study reported that oral administration of powdered *Hericium erinaceus* (lion’s mane) for 16 weeks improved scores on the cognitive function scale compared with participants in the placebo group [26]. Research conducted with mice/rats support that L-ergothioneine may protect against learning/memory deficits [27] and has antidepressant effects [28,29,30]. Together, these findings underscore the need to investigate the effects of mushroom consumption on psychological and cognitive health outcomes in humans using high-quality experimental research. 

We previously reported that adopting a healthy U.S. Mediterranean-style dietary pattern (MED) with 84 g/day *Agaricus bisporus* (white button) or *Pleurotus ostreatus* (oyster) mushrooms improves fasting serum glucose in adults who are overweight or have moderate obesity [31]. Presented here are the results from the secondary objectives of that clinical trial, which were to investigate the effects of consuming the MED diet with 84 g of mushrooms (4 d/week white button and 3 d/week oyster) or a control (breadcrumbs) on indexes of perceived anxiety, depression, mood, well-being, and cognition. Given the paucity of human research assessing these important outcomes, this research is exploratory and will provide novel pilot data to inform future studies.

## 2. Materials and Methods

### 2.1. Experimental Design

The experimental design was a 10-week randomized, parallel, controlled feeding trial. Following a 2-week baseline period, participants were provided with all foods and beverages for a euenergetic, weight maintenance U.S. MED diet with mushrooms (MED-Mushroom) or without (MED-Control) for 8 weeks. Outcome measurements included indexes of anxiety, depression, mood, well-being, and cognition. Before participant recruitment commenced, the Purdue University Institutional Review Board approved the study protocol (IRB 2019-650), and it was registered at Clinicaltrials.gov (NCT04259229). Written consent was obtained from all participants, and they were financially compensated for their time. 

### 2.2. Eligibility Criteria

Male and female (non-pregnant or lactating) volunteers aged 30–69 years without severe or extreme depression (Beck’s Depression Inventory [BDI] score ≤ 30) were recruited from the Greater Lafayette, Indiana, community in the United States of America. Additional inclusion criteria are published [31]: BMI 25.0–34.9 kg/m^2^ (overweight or class 1 obesity classification); non-diabetic; non-smokers; not acutely ill; systolic and diastolic blood pressure < 140/90 mmHg; fasting glucose < 110 mg/dL; fasting serum total cholesterol < 240 mg/dL; triglycerides < 400 mg/dL; and low-density lipoprotein cholesterol < 160 mg/dL. Participants were required to have stable body weight (±3 kg for the previous 3 months), physical activity (previous 3 months), and medication use (previous 6 months). Finally, commitment to consuming only the provided foods/beverages and travel to the clinical research center was required. 

After meeting the study eligibility criteria, participants were randomized to the MED-Control or MED-Mushroom group using the randomization scheme obtained from http://randomization.com (seed 7433; accessed on 10 February 2020). 

### 2.3. Dietary Intervention and Baseline Dietary Assessment

Detailed descriptions of the dietary intervention are published [31]. In brief, participants were provided with all foods and beverages for a euenergetic, weight maintenance U.S. Mediterranean-style diet with mushrooms (MED-Mushrooms) or without (MED-Control) for 8 weeks. Participants received the majority of the intervention foods via a grocery curbside pick-up service. Participants were distributed their weekly fresh white button and oyster mushrooms or control “powder” (breadcrumbs) during their clinic visit weigh-in. At baseline testing, participants received nutrition counseling and menu booklets (7 d rotating menu) containing written instructions regarding food storage and preparation. Instructions for mushroom consumption and preparation were as follows: (1) consume 84 g mushrooms daily with white button 4 d/week and oyster 3 d/week (self-selected variety and day); and (2) mushrooms may be consumed raw or cooked (sauteed or microwaved for 5 min or 30 s, respectively). Participants assigned to the control group (MED-Control) were instructed to mix 1 tsp/d of “powder” (breadcrumbs) into any protocol food. Adherence to the dietary intervention was assessed using self-reported data from weekly menu booklets and was based on consumption of protocol foods/beverages, protocol substitutions, and/or non-protocol food/beverage additions. Supplemental Table S1 outlines weekly food and subgroup amounts of the different energy intake levels compared with the U.S. Mediterranean-style dietary pattern.

### 2.4. Clinical Assessments

Participants reported to the Purdue University Clinical Research Center for the in-clinic fasted (10 h) baseline and post-intervention testing days. Participants completed brain health-related questionnaires, including the General Anxiety Disorder-7 (GAD-7), Beck’s Depression Inventory, Patient Health Questionnaire-9 (PHQ-9), Repeatable Battery for the Assessment of Neuropsychological Status (RBANS), Medical Outcomes Study 36-Item Short Form Health Survey Version 1 (SF36v1), and Profile of Mood States (POMS). Each measure is described in detail below.

#### 2.4.1. Assessment of Symptoms of Generalized Anxiety

Symptoms of generalized anxiety disorder were assessed using the GAD-7, a 7-item self-report measure that asks participants to indicate the frequency at which they have experienced 7 symptoms of generalized anxiety disorder over the past two weeks and to rate how impairing these symptoms have been to their regular functioning. The GAD-7 is commonly used in clinical settings to screen for clinically significant symptoms of generalized anxiety. It has demonstrated good reliability, validity, specificity, and sensitivity [32].

#### 2.4.2. Assessment of Symptoms of Depression

Symptoms of depression were assessed using two complementary self-report measures. The PHQ-9 asks participants to rate the frequency at which they have experienced 9 symptoms of major depressive disorder over the past two weeks and to indicate how impairing these symptoms have been to their daily functioning. The PHQ-9 is a commonly used screening tool for depression in outpatient settings [33] and has previously demonstrated good reliability, validity, sensitivity, and specificity [34]. 

Complementing the PHQ-9, participants also completed Beck’s Depression Inventory (BDI-II) [35]. The BDI-II is a 21-item self-report measure in which participants are asked to report on the intensity (rather than frequency, as with the PHQ-9) of depressive symptoms over the past two weeks. The BDI-II has good psychometric properties in clinical and non-clinical samples [36]. 

#### 2.4.3. Assessment of Mood

Mood was assessed using the Profile of Mood States Short Form (POMS-SF) [37]. The POMS is a 37-item self-report survey that presents one mood-related adjective at a time and asks participants to rate how much they have felt that adjective in the past week, including today. Ratings are measured on a Likert-type scale that ranges from “0-not at all” to “4-Extremely”. The POMS-SF includes six sub-scales (tension, depression, anger, fatigue, confusion, and vigor), each of which is calculated by summing the participant’s ratings of each of the scale’s constituent adjectives. The anger subscale was removed from analyses due to a technical error that occurred during data collection. The POMS-SF exhibits good psychometric properties and is a commonly used measure of general psychological distress [38].

#### 2.4.4. Assessment of Perceptions of Health 

Self-perceptions of health were also assessed prior to and following the diet intervention. To assess this, the Medical Outcomes Study 36-item Short-Form Health Survey (SF-36v1) was used. The SF-36v1 is a self-report survey that asks participants to rate their relative agreement with statements regarding their physical health. All items are rated on Likert-type scales or as yes/no responses. When scored, the SF-36v1 contains 8 scales: psychical functioning, role limitations due to physical health, role limitations due to emotional problems, energy/fatigue, emotional well-being, social functioning, pain, and general health [39]. It has been shown to be a reliable and valid measure of perceptions of health and has previously been used among clinical populations and in intervention studies [40,41,42].

#### 2.4.5. Neuropsychological Assessment

Neuropsychological assessment was conducted by trained research assistants using the Repeatable Battery for the Assessment of Neuropsychological Status (RBANS) [43,44]. As the name implies, this neuropsychological battery was designed to include multiple versions in which the participants complete the same tasks but with different specific items (e.g., participants are asked to copy a figure on each of the versions, but the actual figure presented is different between versions, though matched for difficulty), such that any practice effects are minimized. For this reason, the RBANS is an ideal tool to use for clinical trials in which participant’s cognitive abilities are being assessed at multiple time points. The RBANS takes approximately 30 min to administer and contains 12 subtests, which together measure immediate and delayed memory, visuospatial/constructional capacity, attention, and language. This validated measure has excellent psychometric properties, including good test-retest reliability, resistance to practice effects, and construct validity [45]. 

### 2.5. Statistical Analysis

To reduce risk of bias and ensure accuracy, all outcome data were entered independently by two laboratory technicians and cross-checked by the study coordinator. Data were analyzed in accordance with a pre-specified plan in which analysts remained blinded to the intervention status until all analyses were completed. Statistical analyses were conducted using SPSS (version 29.0 for Windows, IBM Corp., Armonk, NY, USA) [46]. Our primary analyses were used to: (1) compare baseline values (e.g., age, sex, race/ethnicity, and all outcome variables) of the MED-Control and MED-Mushroom groups using *t*-tests and chi-squared tests to ensure demographic matching; (2) evaluate the Group × Time interaction (changes in MED-Mushroom group compared with changes in MED-Control group) using a 2 × 2 ANOVA test with a between-subjects factor of Group; and (3) investigate the main effect of time using a 2 × 2 ANOVA test with a within-subjects factor of Time (pre vs. post). Effect sizes for each outcome were assessed using Cohen’s d (Supplemental Table S2). Results are presented as arithmetic means ± standard errors of the mean (SEM), unless stated otherwise. Statistical significance was set at *p* <0.05. Consistent with the parent study [31], formal power calculations were not completed when designing this exploratory study. Instead, a sample size of *n* = 30 participants per group was chosen to align with the criteria considered by the 2015 Dietary Guidelines Advisory Committee in creating future *Dietary Guidelines for Americans* [47].

## 3. Results

### 3.1. Participant Characteristics

**Figure 1:**
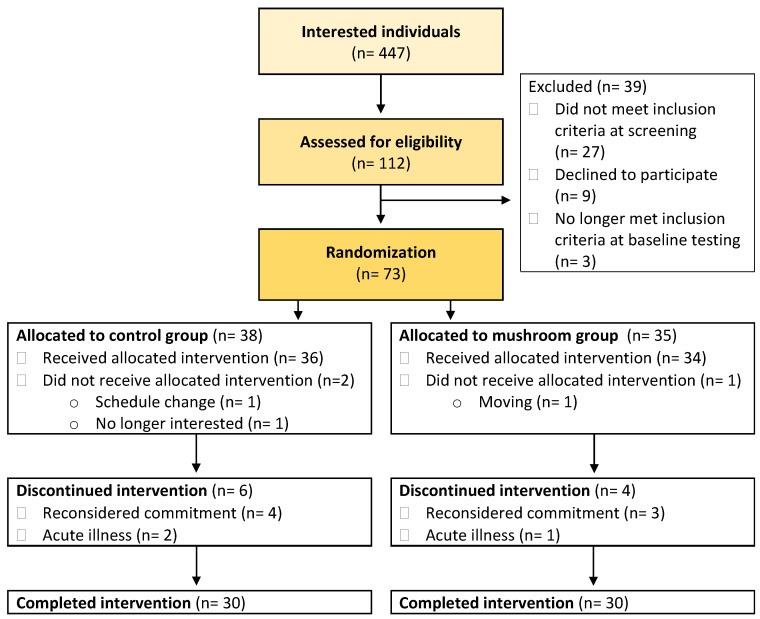
Participant flow CONSORT diagram.

Sixty participants completed the 8-week intervention (Figure 1). At baseline, most participants (*n* = 52, 87%) had normal perceived levels of depression symptoms as assessed via the Beck’s Depression Inventory questionnaire (four participants were taking antidepressant medication) (Table 1). Three participants were categorized as having mild mood disturbance, two had borderline clinical depression symptoms (one was taking antidepressant medication), two had moderate depression symptoms (one was taking antidepressant medication), and one had severe depression symptoms and was taking antidepressant medication. At baseline, there were no differences observed between groups for any outcome except for the SF-36v1 subdomains for emotional role limitations and pain, in which the MED-Control group started with lower levels (higher symptoms; *p* = 0.04 at baseline).

### 3.2. Adherence to the Dietary Intervention

Self-reported data from weekly menu booklets across the intervention period was used to evaluate adherence to the dietary intervention. Across the 8-week intervention, the average adherence to consuming the dietary intervention foods was 92%. More information about the dietary intervention and dietary adherence is published [31].

### 3.3. Anxiety and Depression

There were no changes in anxiety after consumption of the MED diet, assessed via the Generalized Anxiety Disorder-7 questionnaire. Post-intervention Beck’s Depression Inventory scores trended towards an improvement (lowered; Time *p* = 0.084), after consumption of the MED diet, with or without mushrooms. There were no changes in the other measure of depression (Patient Health Questionnaire-9) (Table 2). 

### 3.4. Mood

Consumption of the MED diet, with or without mushrooms, improved (increased) vigor/activity, assessed via the Profile of Mood States (Time *p* = 0.026). There were no changes in other sub-scales of mood including depression, confusion, tension, or fatigue with the consumption of the MED diet (Table 3). 

### 3.5. Perceptions of Health

Consumption of the MED-Control diet improved symptoms of pain (higher score indicates lower symptoms) via the Medical Outcomes Study 36-Item Short Form Health Survey Version 1 (SF-36v1) (Time × Group *p* = 0.044). This finding should be interpreted with caution as the MED-Control group began the study with higher levels of pain than the MED-Mushroom group (MED-Control 77.6 ± 2.69 vs. MED-Mushroom 85.7 ± 3.03; baseline *t*-test *p* = 0.04). There were no changes in other measures of perceived quality of life via the SF-36v1 (Table 4).

### 3.6. Neuropsychological Function

Consumption of the MED diet, with or without mushrooms, improved both subtests of immediate memory (list learning and story memory; Time *p* < 0.005). There were mixed effects for other domains of neuropsychological function. Consumption of the MED diet worsened one subtest of the visuospatial/constructional domain (figure copy; Time *p*= 0.004), but there was no change in the line orientation test. In contrast, consumption of the MED diet improved one subtest of the language domain (picture naming; Time *p* < 0.001), but there was no change in semantic fluency. Regarding the delayed memory domain, consumption of the MED-Control diet improved list recall (mean change 1.37 ± 0.38; Time × Group *p* = 0.005). Consumption of the MED diet, with or without mushrooms, improved story recall (Time *p* < 0.001). There were no changes in the other subtests of the delayed memory domain (list recognition and figure recall). There were no changes in either subtest of the attention domain (digit span or coding) (Table 5).

## 4. Discussion

To the best of our knowledge, this study is the first randomized controlled trial assessing the effects of consuming whole, dietary mushrooms concurrent with adopting a rigorously controlled MED-style dietary intervention on indexes of brain health. Unique to most RCTs regarding mushrooms and brain health, our study integrated a full feed study design with the addition of whole, dietary mushrooms. Given the paucity of human research on this important topic at the time of this study’s conceptualization in 2019, this study was exploratory. More research has been published since then to inform the impact of mushrooms on brain health.

Our findings, indicating that one serving/day of white button/oyster mushrooms for 8 weeks with a healthy MED-style dietary pattern does not influence anxiety, depression, mood, and subjective well-being outcomes, are mostly inconsistent with previous research. Previous research indicates consumption of mushroom capsules, including lion’s mane, improves symptoms of anxiety, stress, and depression [48,49]. Investigators from two separate studies indicate that the consumption of 1.8 g/day of dried lion’s mane capsules [48] or cookies containing dried lion’s mane powder (2 g/d total, 0.5 g/cookie) [49] for 4 weeks reduced rates of anxiety, stress, and depression in healthy adults. Similar to our findings, authors of another study, including healthy older adults who consumed vitamin D-enriched mushroom capsules twice daily for 6 months, indicated no improvements in any mood outcomes (assessed via questionnaires such as the Positive and Negative Affect Scale [PANAS], the 21-item Depression, Anxiety, and Stress Scale [DASS-21], and the General Happiness Scale) [50]. Combined, these studies generally indicate that mushrooms positively influence these brain health outcomes, but the heterogeneity in the study design makes it difficult to compare the results with our work. Further explained, mushroom form (e.g., whole vs. capsule), amount, intervention duration (ranging from 4 weeks [48,49] to 6 months [50]), sample population (healthy [48,49,50] and older adults [50]), and method of assessment (i.e., different questionnaires) varied among previous research. Regarding mushroom form and amount, previous research often includes the use of dried/powdered mushrooms. Given that fresh white button and oyster mushrooms contain around 90–92% water [51,52], we estimate our 84 g serving to be approximately 7–8 g dry weight. While the use of fresh mushrooms is a novel component of our work, a comparison of results should consider this aspect. Taken together, the aforementioned heterogeneity in study designs limits our ability to fully deduce why our results are mostly inconsistent with previous research.

The results from the present study indicate mushroom consumption does not influence cognitive outcomes, which is partly consistent with previous research. Investigators from one study reported no changes in cognitive function (assessed via the Computerized Mental Performance Assessment System [COMPASS]) after daily consumption of lion’s mane powder capsules (1.8 g/day) for 4 weeks in healthy adults [48]. Conversely, evidence from a 16-week study among individuals with cognitive impairment indicates consumption of powdered lion’s mane (four 250 mg tablets, three times per day) improved cognitive function (assessed via the Revised Hasegawa Dementia Scale [HDS-R]) compared with the baseline. However, this improvement was reduced after discontinuation of the intervention [26]. Additionally, investigators from one study that included consumption of 3.2 g/day of lion’s mane powder capsules for 12 weeks reported improvements in overall cognitive function (assessed via the Mini Mental State Examination [MMSE]) in healthy older adults. The MMSE is primarily used in dementia diagnoses and measures functions such as orientation, concentration, attention, verbal memory, naming, and visuospatial skills. In contrast to our findings, there were no changes in the quality of memory or short-term memory subdomains [53]. As previously mentioned, the heterogeneity in study design (e.g., mushroom species, form, doses, intervention periods, study population, and cognitive measures) among previous research and the present study creates challenges for comparing results.

Independent of mushroom intake, limited clinical and epidemiological evidence suggests that the MED diet positively impacts cognitive function and depression symptoms. Consistent with our results (consumption of a MED diet improves immediate memory), evidence from an epidemiological study among 51,529 middle-aged and older men indicates a positive association between high adherence to a MED diet and improvements in cognitive function [54]. Additionally, authors of a meta-analysis including 19 studies from 9 different countries concluded that the MED diet and similar diets (e.g., low carbohydrate and/or high consumption of olive oil) positively impact cognitive function, including processing speed, executive function, and language skills in cognitively healthy adults [55]. Regarding depression symptoms, investigators of an RCT reported that consumption of a MED-style diet intervention for 24 weeks reduced depressive symptoms compared with the control group in adults diagnosed with moderate to severe depression [56]. The inconsistency in findings between this RCT and our work may be due to the longer intervention period (24 weeks vs. 8 weeks) and study population (participants had depressive symptoms for at least 2 months compared with our mentally healthy population).

Regarding potential limitations of our study, as previously mentioned, this exploratory study did not include formal power calculations at the time of its design. Instead, a sample size of *n* = 30 participants per group was chosen to align with the criteria considered by the 2015 Dietary Guidelines Advisory Committee in creating future *Dietary Guidelines for Americans*. Future studies evaluating the effects of consuming mushrooms on indexes of brain health should be adequately powered. Another consideration is that we were unable to blind participants in the mushroom group, given they were provided with fresh mushrooms throughout the eight-week intervention period.

Strengths of this research include the use of a randomized, controlled trial, in which all foods and beverages were provided to participants. Although relatively short in duration, there was a high adherence to the dietary intervention (92% self-reported mean adherence to consuming the protocol foods) and a low participant drop-out percentage (<18%). Additionally, participants avoided other interventions such as changes in physical activity or the use of dietary supplements throughout the intervention period [31]. While the study coordinator was responsible for distributing some intervention foods (i.e., fresh mushrooms or breadcrumbs) and overseeing in-clinic test visits, participants were provided with a quiet space to fill out the subjective questionnaires and were proctored by laboratory technicians who were unaware of participant assignment. Additional steps were taken to reduce risk of bias including de-identification of data, double data entry, and cross-checking. Finally, data analysts, who had no role in data collection, were blinded to group assignments until the data analysis was completed.

Considering the modest and predominantly neutral responses in brain health indexes observed in this mentally healthy population of middle-aged and older adults, future investigators may consider assessing these brain health outcomes in individuals diagnosed with depression or declining cognition. Additional considerations include the use of other mushroom species, including lion’s mane, which are shown to positively impact multiple brain health outcomes. Future research could extend the intervention period beyond 8 weeks to determine if different results are obtained compared with the current study and may include titrated mushroom doses to assess if there is a dose–response relationship. Finally, future investigations should assess the influence of the food matrix and food processing on the bioavailability of mushroom-derived compounds [57,58].

## 5. Conclusions

In conclusion, the adoption of a healthy MED-style dietary pattern, with or without 84 g per day (one serving) of whole white button and oyster mushrooms, for 2 months improved vigor/activity and immediate memory among cognitively healthy middle-aged and older adults without severe depression.

## Figures and Tables

**Table 1:** Baseline demographic and depression characteristics.

Demographic Characteristics	MED-Control (*n* = 30)	MED-Mushroom (*n* = 30)	Total (*n* = 60)
Age at randomization (years) Female, n (%) White, n (%)	44 ± 12	47 ± 13	46 ± 12
	20 (60)	16 (60)	36 (60)
	22 (73)	19 (63)	41 (68)
Hispanic or Latinx, n (%) Asian, n (%)	5 (17)	5 (17)	10 (17)
	2 (7)	5 (17)	7 (12)
Black, n (%) Other (not specified), n (%) Weight (kg) BMI (kg/m^2^)	1 (3)	0 (0)	1 (2)
	0 (0)	1 (3)	1 (2)
	82.6 ± 15.2	84.7 ± 14.3	83.7 ± 14.7
	28.4 ± 2.76	28.3 ± 2.91	28.3 ± 2.84
Depression Characteristics			
Beck’s Depression Inventory (0–63)	7.0 ± 8.96	4.7 ± 4.82	5.8 ± 7.23
Levels of depression, n (%)			
Normal (1–10)	24 (80)	28 (93)	52 (87)
Mild mood disturbance (11–16)	3 (10)	0 (0)	3 (5)
Borderline clinical depression (17–20)	0 (0)	2 (7)	2 (3)
Moderate depression (21–30)	2 (7)	0 (0)	2 (3)
Severe depression (31–40) ^1^	1 (3)	0 (0)	1 (2)
Extreme depression (>40)	0 (0)	0 (0)	0 (0)

Values are the means ± SD. At baseline, no statistically significant differences were observed between groups for any of the outcomes listed in this table. ^1^ One participant had a BDI score categorized as “severe depression” at baseline testing but not at their screening qualification visit (BDI score = 10 at screening qualification visit).

**Table 2:** Changes in indexes of perceived anxiety and depression after 8 weeks of following a MED diet with or without mushrooms.

	MED-Control			MED-Mushroom			*p*-Values	
Outcome (au)	Baseline	Post	Change	Baseline	Post	Change	Time	Time × Group
Generalized Anxiety Disorder-7 (0–21)	3.1 ± 0.67	2.8 ± 0.69	−0.27 ± 0.50	2.1 ± 0.51	2.0 ± 0.61	−0.07 ± 0.34	0.581	0.74
Beck’s Depression Inventory (0–63)	7.0 ± 1.64	6.2 ± 1.38	−0.77 ± 0.80	4.7 ± 0.88	3.4 ± 0.91	−1.23 ± 0.84	0.088	0.687
Patient Health Questionnaire-9 (0–27)	4.5 ± 0.86	3.6 ± 0.79	−0.87 ± 0.57	2.8 ± 0.69	2.8 ± 0.84	0.00 ± 0.50	0.257	0.257

Values are the means ± SEM (*n* = 60 total, *n* = 30/group). At baseline, no statistically significant differences were observed between groups for any of the outcomes listed in this table. Abbreviations: au: arbitrary units.

**Table 3:** Changes in mood states after 8 weeks of following a MED diet with or without mushrooms.

	MED-Control			MED-Mushroom			*p*-Values	
Mood ^1^ (au)	Baseline	Post	Change	Baseline	Post	Change	Time	Time × Group
Depression (0–32)	0.2 ± 0.06	0.2 ± 0.08	0.00 ± 0.08	0.2 ± 0.08	0.2 ± 0.12	0.03 ± 0.07	0.779	0.841
Vigor (0–24)	1.4 ± 0.17	1.5 ± 0.18	0.13 ± 0.13	1.7 ± 0.21	2.0 ± 0.20	0.29 ± 0.13	0.026	0.41
Confusion (0–20)	0.4 ± 0.07	0.3 ± 0.07	−0.07 ± 0.07	0.3 ± 0.09	0.4 ± 0.13	0.05 ± 0.07	0.844	0.267
Tension (0–24)	0.4 ± 0.08	0.3 ± 0.09	−0.06 ± 0.06	0.3 ± 0.10	0.3 ± 0.12	0.00 ± 0.07	0.511	0.543
Fatigue (0–20)	0.7 ± 0.16	0.6 ± 0.17	−0.05 ± 0.13	0.4 ± 0.12	0.6± 0.19	0.21 ± 0.13	0.411	0.165

Values are the means ± SEM (*n* = 60 total, *n* = 30/group). At baseline, no statistically significant differences were observed between groups for any of the outcomes listed in this table. ^1^ Measured by the Profile of Mood States questionnaire. Abbreviations: au: arbitrary units.

**Table 4:** Changes in perceived quality of life after 8 weeks of following a MED diet with or without mushrooms.

	MED-Control			MED-Mushroom			*p*-Values	
SF-36v1 Scale ^1^ (0–100 au)	Baseline	Post	Change	Baseline	Post	Change	Time	Time × Group
Physical functioning	89.9 ± 2.02	91.0 ± 1.60	1.15 ± 1.69	88.7 ± 2.48	86.3 ± 3.59	−2.33 ± 3.10	0.741	0.333
Physical RL	90.5 ± 3.81	98.3 ± 1.20	7.76 ± 3.94	94.2 ± 3.73	91.7 ± 4.04	−2.50 ± 4.04	0.356	0.075
Emotional RL	69.0 ± 7.75	75.9 ± 7.00	6.90 ± 7.47	88.9 ± 4.88	84.4 ± 5.47	−4.44 ± 4.72	0.781	0.201
Energy/fatigue	57.1 ± 4.25	61.4 ± 4.55	4.31 ± 2.92	62.3 ± 3.97	61.7 ± 4.64	−0.67 ± 2.72	0.365	0.217
Emotional well-being	75.2 ± 3.37	76.0 ± 3.18	0.83 ± 2.73	80.4 ± 2.89	79.9 ± 3.13	−0.53 ± 1.93	0.93	0.684
Social functioning	83.2 ± 4.22	84.5 ± 4.19	1.29 ± 3.24	89.6 ± 3.06	92.1 ± 2.58	2.50 ± 2.35	0.345	0.763
Pain	77.6 ± 2.69	82.0 ± 2.95	4.40 ± 1.88	85.7 ± 3.03	83.8 ± 3.37	−1.92 ± 2.41	0.422	0.044
General health	68.8 ± 3.16	72.1 ± 2.41	3.28 ± 2.38	76.2 ± 2.95	76.5 ± 2.99	0.33 ± 2.06	0.256	0.353

Values are the means ± SEM. Baseline: *n* = 60 total, *n* = 30/group; post-intervention: *n* = 59 total, *n* = 29 for MED-Control and *n* = 30 for MED-Mushroom. At baseline, no statistically significant differences were observed between groups for any of the outcomes listed in this table except for emotional role limitations and pain, in which the MED-Control group started with lower levels (higher symptoms; *p* = 0.04 at baseline). ^1^ Measured by the Medical Outcomes Study 36-Item Short Form Health Survey Version 1 (SF-36v1). Abbreviations: au: arbitrary units; RL: role limitations.

**Table 5:** Changes in neuropsychological indexes after 8 weeks of following a MED diet with or without mushrooms.

		MED-Control			MED-Mushroom			*p*-Values	
RBANS Subtests ^1^ (au)		Baseline	Post	Change	Baseline	Post	Change	Time	Time × Group
Immediate Memory	List learning (0–40)	28.2 ± 0.76	30.3 ± 0.64	2.03 ± 0.76	28.9 ± 0.76	30.7 ± 0.76	1.80 ± 0.74	<0.001	0.826
	Story memory (0–24)	16.3 ± 0.71	17.3 ± 0.50	1.03 ± 0.65	17.2 ± 0.50	18.1 ± 0.66	0.93 ± 0.59	0.029	0.91
Visuospatial/Constructional	Figure copy (0–20)	15.8 ± 0.57	14.4 ± 0.58	−1.40 ± 0.59	15.9 ± 0.55	14.9 ± 0.53	−1.00 ± 0.55	0.004	0.622
	Line orientation (0–20)	17.2 ± 0.47	17.4 ± 0.49	0.17 ± 0.40	17.8 ± 0.40	17.2 ± 0.50	−0.67 ± 0.47	0.422	0.183
Language	Picture naming (0–10)	8.6 ± 0.32	9.5 ± 0.15	0.90 ± 0.24	8.5 ± 0.32	9.6 ± 0.12	1.10 ± 0.25	<0.001	0.564
	Semantic fluency (0–40)	22.5 ± 1.01	22.2 ± 0.99	−0.33 ± 1.00	21.8 ± 1.22	22.1 ± 1.03	0.30 ± 0.94	0.981	0.646
Attention	Digit span (0–16)	10.6 ± 0.47	10.8 ± 0.59	0.23 ± 0.51	11.3 ± 0.46	12.2 ± 0.54	0.97 ± 0.40	0.068	0.26
	Coding (0–89)	51.5 ± 1.59	52.4 ± 1.75	0.83 ± 1.16	53.3 ± 1.29	54.8 ± 1.55	1.47 ± 1.33	0.197	0.721
Delayed Memory	List recall (0–10)	6.3 ± 0.38	7.7 ± 0.25	1.37 ± 0.38	7.0 ± 0.36	7.1 ± 0.36	0.13 ± 0.35	0.005	0.019
	List recognition (0–20)	19.7 ± 0.10	19.7 ± 0.13	−0.03 ± 0.17	19.6 ± 0.16	19.6 ± 0.13	0.07 ± 0.10	0.864	0.609
	Story recall (0–12)	8.7 ± 0.42	9.6 ± 0.32	0.97 ± 0.28	9.2 ± 0.38	10.0 ± 0.38	0.80 ± 0.39	<0.001	0.731
	Figure recall (0–20)	12.0 ± 0.69	11.8 ± 0.66	−0.20 ± 0.58	13.4 ± 0.73	13.5 ± 0.55	0.10 ± 0.60	0.905	0.721

Values are the means ± SEM (*n* = 60 total, *n* = 30/group). At baseline, no statistically significant differences were observed between groups for any of the outcomes listed in this table. ^1^ Measured by the Repeatable Battery for the Assessment of Neuropsychological Status (RBANS). Abbreviation: au: arbitrary units.

## Data Availability

The original contributions presented in the study are included in the article/Supplementary Materials, further inquiries can be directed to the corresponding author.

## Supplementary Materials

The following supporting information can be downloaded at: https://www.mdpi.com/article/10.3390/foods13152319/s1, Table S1: Servings of food groups in the USDA Healthy Mediterranean-Style Dietary Pattern and Study Diets, Table S2: Cohen’s d effect size by outcome.
