# Supplementary material for: Effects of Consuming White Button and Oyster Mushrooms within a Healthy Mediterranean-Style Dietary Pattern on Changes in Subjective Indexes of Brain Health or Cognitive Function in Healthy Middle-Aged and Older Adults
Source: Foods. 2024 Jul 23;13(15):2319. doi: 10.3390/foods13152319 (PMC11311880; doi:10.3390/foods13152319)
Supplement: Supplementary file 1 [file foods-13-02319-s001.zip › foods-3099982-supplementary.pdf]

Uffelman, CN. Effects of Consuming White Button and Oyster Mushrooms Within a Healthy Mediterranean-Style Dietary Pattern on Changes in Subjective Indexes of Brain Health or Cognitive Function in Healthy Middle-Aged and Older Adults

Supplemental Table S1. Servings of Food Groups in the USDA Healthy Mediterranean-Style Dietary Pattern and Study Diets

**Dietary Pattern Energy Level (kcal/d)**	**2,000**		**2,400**		**2,800**	
**Food Group**	**USDA Med-HDP**[a]	**Study Med-HDP**[b]	**USDA Med-HDP**	**Study Med-HDP**	**USDA Med-HDP**	**Study Med-HDP**
**Vegetables (total), c-eq/day**	2.5[c]	2.5	3	3	3.5	3.5
Dark-green (c-eq/wk)	1.5	3	2	3.5	2.5	4
Red and orange (c-eq/wk)	5.5	5.5	6	6.5	7	7.5
Legumes (beans and peas) (c-eq/wk)	1.5	1.5	2	2	2.5	2.5
Starchy vegetables (c-eq/wk)	5	5	6	5	7	7.5
Other vegetables (c-eq/wk)	4	3	5	4	5.5	4
**Fruits (total), c-eq/day**	2.5	2	2.5	2	3	2.5
**Grains (total), oz-eq/day**	6	6	8	7.5	10	9.5
Whole grains (oz-eq/day)	3	3.5	4	4.5	5	5
Refined grains (oz-eq/day)	3	2.5	4	3.5	5	4
**Dairy, c-eq/day**	2	2	2.5	2	2.5	2.5
**Protein foods, oz-eq/day**	6.5	6.5	7.5	7.5	8	8.5
Seafood (oz-eq/wk)	15	15	16	16	17	17
Meats, poultry, eggs (oz-eq/wk)	26	26	31	32.5	33	34.5
Nuts, seeds, soy (oz-eq/wk)	5	4	5	5	6	6.5
**Oils, g/day**	27	21.5	31	24	36	31.5
**Limit on calories for other uses**	260	116	300	113	350	117

[a]USDA healthy Mediterranean-style eating pattern recommended amounts of food from each food group following Appendix 4 of the 2015-2020 Dietary Guidelines for Americans.

[b]Average daily or weekly amounts of foods from each food group/subgroup in the control dietary pattern. Participants in the mushroom group consumed more total vegetables (84 g/d or ~1 c-eq/day) not reflected here. Values listed for the study Med-HDP are rounded to the nearest half number.

[c]Daily amount of food from each group is listed. Subgroup amounts for vegetable and protein foods are per week.

Abbreviations: c-eq/day: cup-equivalence per day; c-eq/wk: cup-equivalence per week; g/day: grams/day; Med-HDP: Mediterranean-Style Healthy Dietary Pattern; oz-eq/day: ounce-equivalence per day; USDA: United States Department of Agriculture

Uffelman, CN. Effects of Consuming White Button and Oyster Mushrooms Within a Healthy Mediterranean-Style Dietary Pattern on Changes in Subjective Indexes of Brain Health or Cognitive Function in Healthy Middle-Aged and Older Adults

Supplemental Table S2. Cohen's d effect size by outcome

Outcome (au)		n	Paired Difference		Standardizer[1]	Point Estimate[2]	95% CI Lower	95% CI Upper
			Mean	SD				
**Generalized Anxiety Disorder-7 (0-21)**		60	-0.17	2.31	3.44	-0.05	-0.22	0.13
**Beck's Depression Inventory (0-63)**		60	-1.00	4.44	6.95	-0.14	-0.31	0.02
**Patient Health Questionnaire-9 (0-27)**		60	-0.43	2.94	4.39	-0.10	-0.27	0.08
**Mood (Profile of Mood States)**								
	Depression (0-32)	60	0.01	0.40	0.52	0.03	-0.17	0.23
	Vigor (0-24)	60	0.21	0.71	1.05	0.20	0.02	0.38
	Confusion (0-20)	60	-0.01	0.39	0.52	-0.02	-0.21	0.17
	Tension (0-24)	60	-0.03	0.35	0.55	-0.05	-0.22	0.11
	Anger (0-28)	60	0.01	0.46	0.37	0.02	-0.31	0.35
	Fatigue (0-20)	60	0.08	0.72	0.91	0.08	-0.12	0.29
**SF-36v1 Scale (0-100)**								
	Physical functioning	59	-0.62	13.70	14.04	-0.04	-0.30	0.21
	Physical role limitations	59	2.54	22.12	18.66	0.14	-0.17	0.45
	Emotional role limitations	59	1.13	33.88	35.05	0.03	-0.22	0.28
	Energy/fatigue	59	1.78	15.39	23.80	0.08	-0.09	0.24
	Emotional well-being	59	0.14	12.69	17.07	0.01	-0.19	0.20
	Social functioning	59	1.91	15.19	19.53	0.10	-0.11	0.30
	Pain	59	1.19	12.10	16.61	0.07	-0.12	0.26
	General health	59	1.78	12.06	16.04	0.11	-0.09	0.31
**RBANS Subtests**								
**Immediate Memory**	List learning (0-40)	60	1.92	4.07	3.99	0.48	0.20	0.76
	Story memory (0-24)	60	0.98	3.37	3.29	0.30	0.03	0.57

Uffelman, CN. Effects of Consuming White Button and Oyster Mushrooms Within a Healthy Mediterranean-Style Dietary Pattern on Changes in Subjective Indexes of Brain Health or Cognitive Function in Healthy Middle-Aged and Older Adults

Outcome (au)		n	Paired Difference		Standardizer[1]	Point Estimate[2]	95% CI Lower	95% CI Upper
			Mean	SD				
Visuospatial/ Constructional	Figure copy (0-20)	60	-1.20	3.11	3.03	-0.40	-0.67	-0.12
	Line orientation (0-20)	60	-0.25	2.41	2.55	-0.10	-0.34	0.15
Language	Picture naming (0-10)	60	1.00	1.33	1.78	0.56	0.34	0.78
	Semantic fluency (0-40)	60	-0.02	5.28	5.82	0.00	-0.24	0.23
Attention	Digit span (0-16)	60	0.60	2.51	2.94	0.20	-0.02	0.43
	Coding (0-89)	60	1.15	6.77	8.58	0.13	-0.07	0.34
Delayed Memory	List recall (0-10)	60	0.75	2.06	1.89	0.40	0.11	0.69
	List recognition (0-20)	60	0.02	0.75	0.71	0.02	-0.25	0.30
	Story recall (0-12)	60	0.88	1.85	2.07	0.43	0.18	0.67
	Figure recall (0-20)	60	-0.05	3.22	3.70	-0.01	-0.24	0.21

[1]The denominator used in estimating the effect sizes.

[2]Cohen's d uses the sample standard deviation of the mean difference adjusted by the correlation between measures.

Abbreviations: au: arbitrary units; CI: confidence interval; RBANS: Repeatable Battery for the Assessment of Neuropsychological Status
